# Mid‐term results of reconstruction rings used in combination with modular tantalum augments for Paprosky type III acetabular defects in revision hip arthroplasty

**DOI:** 10.1002/jeo2.70489

**Published:** 2025-11-05

**Authors:** David Spranz, Lisa‐Marie Müller, Raphael Trefzer, Pit Hetto, Timo Nees, Tilman Walker, Tobias Reiner

**Affiliations:** ^1^ Department of Orthopaedics Heidelberg University Hospital Heidelberg Germany

**Keywords:** individual acetabular defect reconstruction, modularity, reinforcement ring, revision total hip arthroplasty, tantalum trabecular metal augments

## Abstract

**Purpose:**

The combined use of reconstruction rings and modular trabecular metal (TM) augments can be a viable individual treatment in selected patients with large severe acetabular bone defects. However, clinical data on the outcome of this surgical technique is limited. This study aimed to evaluate mid‐term results of reconstruction rings used in combination with modular TM augments for severe acetabular defects in revision hip arthroplasty.

**Methods:**

We retrospectively reviewed 23 patients with Paprosky type III A or B acetabular defects who underwent revision surgery using a reconstruction ring with a cemented cup in combination with a modular TM augment. 16 patients had type III A defects and 7 patients suffered from type III B defects. Clinical outcome was assessed using patient‐reported outcome scores (PROMs). CT scans were used to assess preoperative bone loss and plain radiographs were used to determine postoperative implant migration. Blood tantalum concentrations were measured at latest follow‐up and compared to a control group of patients without metal implants.

**Results:**

18 patients could be contacted. 9 patients underwent a complete clinical and radiological follow‐up examination as well as a blood test. The cumulative survival rate at 7.4 years with the endpoint ‘acetabular component revision for any reason’ was 86.7% (95% confidence interval 56%–96%). At the most recent follow‐up two patients (9%) had undergone revision surgery due to aseptic loosening of the acetabular construct. Three patients showed radiological signs of loosening of the reconstruction ring without clinical symptoms. The PROMs improved significantly to the latest follow‐up. Blood tantalum concentrations were elevated in the study group (0.06 µg/L) compared to controls (0.002 µg/L) (*p* < 0.001).

**Conclusions:**

In this study, favourable mid‐term (mean 7.4 years) clinical and radiological outcomes of modular TM augments in combination with a reinforcement ring and cemented revision cups for individual reconstructing major acetabular defects were observed. Aseptic loosening is the main reason for revision, whereby the TM augment was firmly osseointegrated in all cases.

**Level of Evidence:**

Level IV, retrospective case series.

**Abbreviations**
BMI: body mass index
CI: confidence interval
CT: computed tomography
DAIR: debridement, antibiotics, and implant retention
FU: follow‐up
HHS: Harris Hip Score
MCS‐12: Mental Component Summary of the 12‐Item Short Form Health Survey
PCS‐12: Physical Component Summary of the 12‐Item Short Form Health Survey
PE: polyethylene
PJI: periprosthetic joint infection
PROMs: patient‐reported outcome measures
RD: reinforcement device
SD: standard deviation
SF‐12: 12‐Item Short Form Health Survey
THA: total hip arthroplasty
TM: trabecular metal
UCLA: University of California at Los Angeles (Activity Score)

## INTRODUCTION

Total hip arthroplasty (THA) continues to yield excellent long‐term outcomes in terms of pain relief, restoration of mobility and improved function [21, 29]. However, failure modes such as prosthetic dislocation [21], aseptic loosening and periprosthetic joint infection frequently lead to progressive acetabular bone loss, particularly in the revision setting [29]. Addressing advanced periacetabular defects in revision THA is challenging and requires a strategy that ensures durable implant fixation, anatomical restoration of joint biomechanics and reconstitution of the native bone structure [8, 11]. Successful reconstruction of complex acetabular defects and pelvic discontinuities requires careful preoperative planning, specific operative techniques, and a comprehensive understanding of implant design, bone ingrowth potential and bearing materials [23]. Acetabular reinforcement devices (RDs) such as the Contour cage, the Ganz ring, or the Burch–Schneider cage have been widely applied for structural support in revision settings [15]. These RDs may be used in combination with plating of the anterior and/or posterior columns and different types of bone grafting for defect filling [7]. A cemented cup is then usually inserted into this structure. Another solution for the individual management of uncontained structural acetabular defects is the use of modular trabecular metal (TM) implants composed of tantalum alloy. Recent research indicates that tantalum exhibits favourable biocompatibility and outstanding osseointegration characteristics [12, 16, 18, 20, 36]. In situations of massive bone loss, combining modular TM augments with an acetabular RD and a cemented cup may allow both initial mechanical stability and individual reconstruction of the hip centre. However, clinical data supporting this specific construct—TM augments in combination with a reconstruction ring and cemented cup—are scarce. This study aimed to assess the mid‐term clinical and radiological outcomes of a technique combining modular TM augments with acetabular reconstruction rings and cemented cups for the management of severe acetabular defects in revision hip arthroplasty. Secondary objectives included the evaluation of blood tantalum levels and identification of complications associated with this specific construct.

## MATERIALS AND METHODS

### Patient selection and study cohort

We retrospectively analyzed data from a cohort of 23 patients with severe acetabular defects (Paprosky Type IIIa or IIIb) [25] who underwent complex acetabular reconstruction between January 2011 and December 2020. This reconstruction involved the use of a modular TM augment (ZimmerBiomet) in combination with an acetabular reconstruction ring system and either a cemented dual‐mobility cup (AVANTAGE®; ZimmerBiomet) or a cemented Mueller low‐profile polyethylene cup (ZimmerBiomet). The minimum follow‐up period was 2 years. Ethical approval was obtained from the local institutional review board (S‐122/2021), and written informed consent was secured from all study participants. The study was conducted in accordance with the Helsinki Declaration of 1975, as revised in 2013. The classification of acetabular defects was based on conventional X‐rays, computed tomography scans, and operative reports. Preoperative CT scans, conventional radiographs, and operative reports were available for all patients.

### Surgical technique and postoperative management

The revision arthroplasty was performed by 5 senior surgeons in a laminar flow operating room. A lateral approach was used in all patients. First, the original acetabular component was exposed and removed. Curets, osteotomes, and hemispherical reamers were used to debride cement and scarred capsular tissue to fully expose the acetabulum. Hemispherical reamers were used to prepare the acetabular bone and to achieve a well vascularized bone. The trial augment was placed against the deficient host bone and correct sizing was chosen according to the diameter of the last reamer and the height of the defect in order to anatomically reconstruct the acetabular centre of rotation. In cases with an extensive segmental bone defect, modular TM buttress augments were used. The TM augment was then secured to the host bone with 6.5 mm cancellous screws. The number of screws that can be used depends on the size of the implant and ranges from 2 to a maximum of 6 screws for a buttress augment. In all cases, an attempt was made to use as many screws as possible. The flanges of the reconstruction ring were bent and shaped to fit the specific anatomy of the augmented acetabulum. The superior flange was fixed to the iliac bone with cancellous bone screws, and the inferior flanges were fixed to the ischium and/or the pubis. Bone cement was placed in the cage and pressurized to make it exude into the voids of the cage in order to ensure a uniformly cement penetration into the gaps of the cage and between the TM augment and the reconstruction ring. A polyethylene cup or a dual mobility cup was then cemented into the cage with an appropriate anteversion and abduction angle. Patients began training quadriceps femoris strength, hip flexion and hip abduction on the first day after surgery. Partial weight‐bearing with 20 kg was prescribed for 6 weeks after the operation and then gradually switched to full weight‐bearing.

### Clinical and radiographic follow‐up

The clinical evaluation involved the Harris Hip Score (HHS), the UCLA Activity Score and the Short Form‐12 Health Survey (SF‐12 Score) [33].

Acetabular revision was defined as the replacement of one or more components of the acetabulum, including the acetabular reconstruction ring, TM augment, screws, or the cup.

Radiological assessments were performed using standard anteroposterior and lateral hip X‐rays, focusing on the presence of radiolucencies, osteolysis, or migration of the implant components up to the most recent follow‐up. Loosening of the augment/reconstruction ring/cup construct was identified if (1) there was more than 3 mm of migration compared to the early postoperative X‐ray, (2) a progressive radiolucent line was observed at the augment‐bone or cement‐bone interface, or (3) a screw or component fracture was visible on the X‐ray. Two independent orthopaedic surgeons specialized in THA (D.S. and T.R.) evaluated the radiographs.

### Blood examination

For blood examination, a whole blood sample (7.5 mL) was collected from each patient during the follow‐up examination, along with samples from 15 control patients without metal implants. Whole blood was collected, stabilized in sodium heparin‐treated polypropylene tubes, and frozen until analysis. Tantalum concentrations were measured by an accredited laboratory (Geological Institute, University of Heidelberg) using inductively coupled plasma mass spectrometry [27]. The results from the control group have been published in a separate study [30, 31].

### Statistical analysis

All statistical analyses were performed using SPSS® software (version 26.0; IBM Corp.). Statistical significance was defined as *p* < 0.05. Descriptive statistics were calculated as means with standard deviation (SD) and ranges. Preoperative and postoperative outcomes were compared using paired *t*‐tests. Kaplan–Meier survival analysis was performed with revision for any reason as the endpoint.

## RESULTS

### Patient cohort

**Figure 1:**
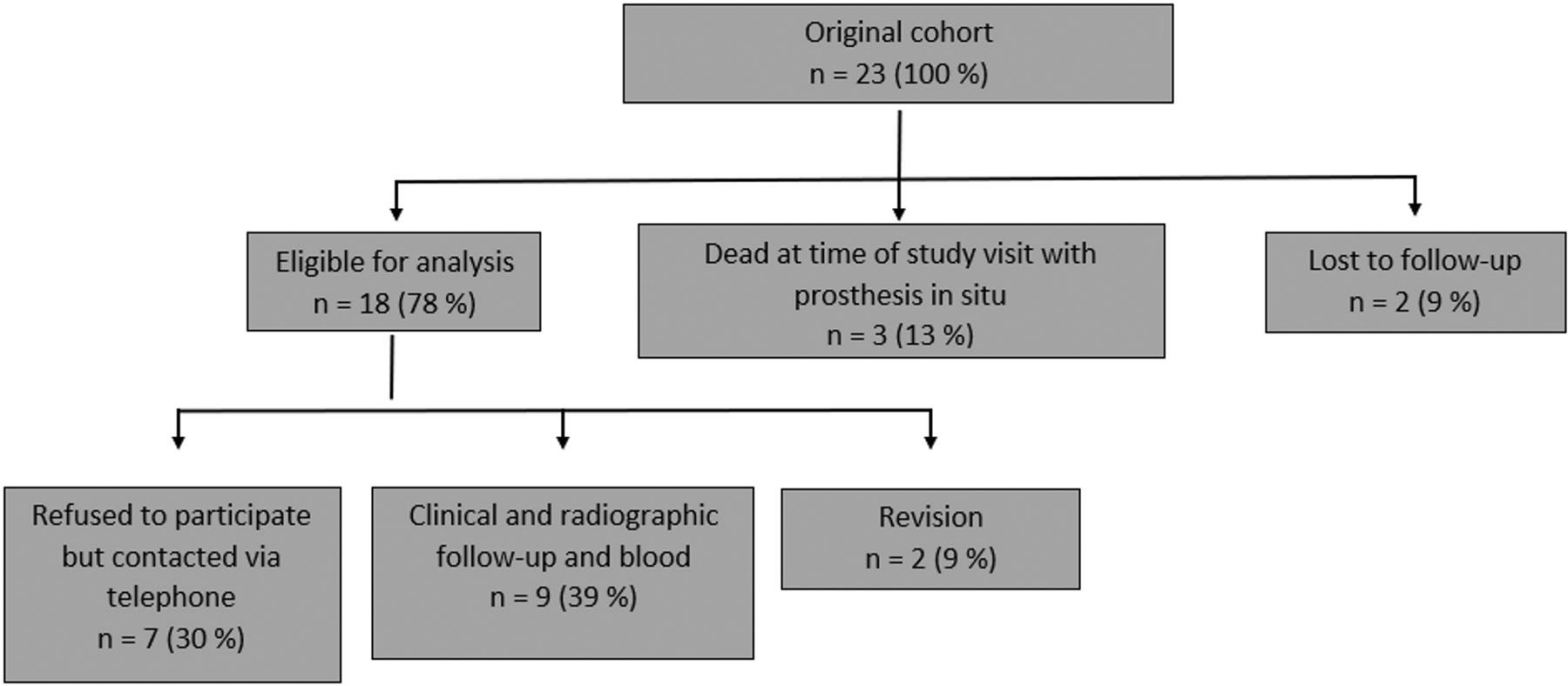
Clinical trial profile and patient flowchart.

Figure 1 illustrates the clinical trial profile and the flow of patients throughout the study. From the initial cohort of 23 patients, 2 patients (9.0%) were lost to follow‐up due to unknown addresses or being in a foreign country. Additionally, 3 patients (13.0%) passed away from unrelated causes while the implant remained in place. Ultimately, 18 patients (78%) were included in the analysis. The average follow‐up duration for this group was 7.4 years (SD 3.0; range 2.0–11.4 years). Among these 18 patients, there were 3 males and 15 females, with a mean age of 64.3 years (range 37–80). The average BMI was 26.7 kg/m² (range 20.5–32.5). Based on the Paprosky classification system [25], 13 cases were categorized as grade IIIa and 5 as grade IIIb. Seven patients (30%) declined clinical and radiological evaluations. These patients were reachable by phone, and all reported no prior revision surgeries. Two patients (2%) underwent acetabular revision surgery. Complete clinical and radiological follow‐up data were available for 9 patients (39.0%) with a mean follow‐up of 7.2 years (SD 3.3; range 2.0–11.4 years). Blood samples from these 9 patients were analyzed for tantalum concentration.

**Figure 2:**
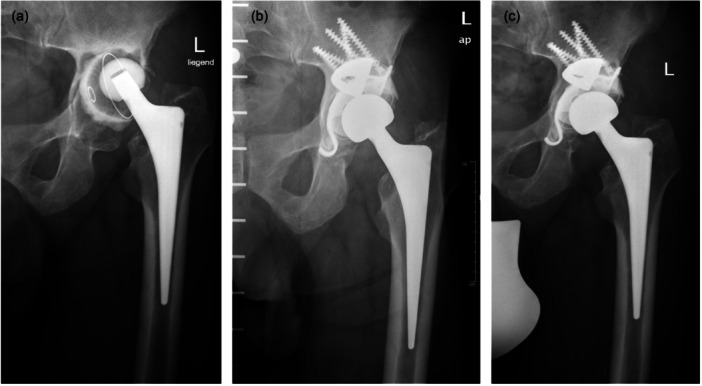
(a) Preoperative anteroposterior radiograph of the hip of a 67‐year‐old patient with severe acetabular bone loss (Paprosky type IIIa) due to aseptic loosening of the cemented cup (a). Anteroposterior radiograph of the same patient postoperative (b) and 10.8 years after surgery (c) using a modular tantalum TM buttress augment in combination with a Ganz reinforcement ring and a cemented PE cup. There are no signs of loosening of one of the components. TM, trabecular metal

The indications for surgery included revision THA due to aseptic loosening of the cup in 16 cases (Figure 2) and two‐stage revision THA for infection in 2 cases.

**Figure 3:**
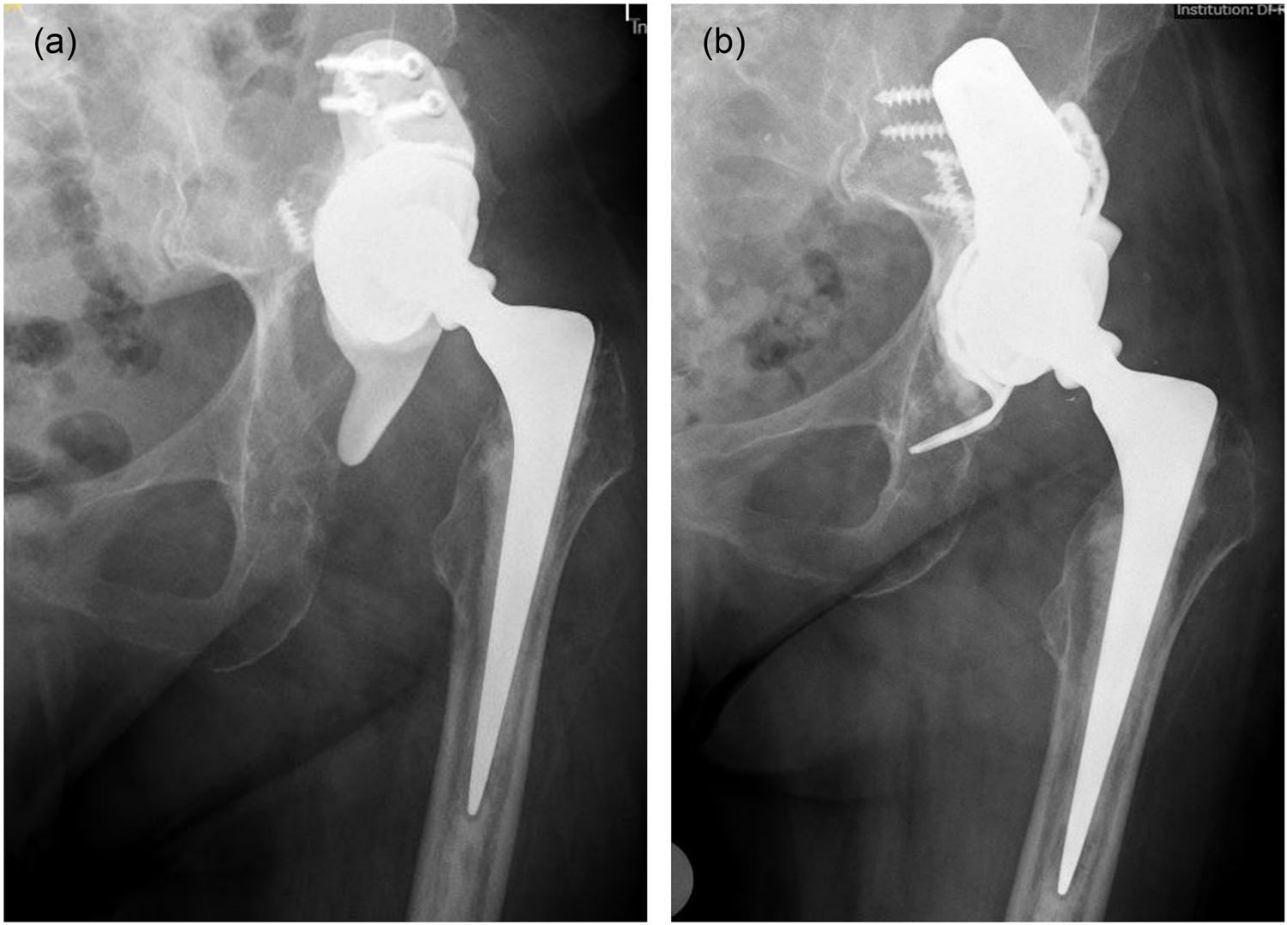
(a) Preoperative anteroposterior radiograph of the hip of an 80‐year‐old patient with severe acetabular bone loss due to aseptic loosening of the cup construct. (b) Anteroposterior radiograph of the same patient postoperative 4.0 years after surgery using a modular tantalum TM buttress augment in combination with a Contourring and a cemented dual‐mobility cup. There are no signs of loosening of one of the components. TM, trabecular metal.

In 16 cases, a TM augment (Figure 2) was used to fill a superior bone void. In 2 cases a TM buttress augment (Figure 3) was used.

The augments were combined with reconstruction cages (in 8 cases Ganz reinforcement ring (ZimmerBiomet; Figure 2), in 9 cases Contourring (Stryker; Figure 3) and in one case Burch–Schneider antiprotrusio cage (ZimmerBiomet) to restore the acetabular wall and reconstruct the anatomical acetabular centre of rotation.

In 7 cases, a cemented dual‐mobility cup (Figure 3) and in 11 cases a cemented polyethylene cup (Figure 2) was used.

### Survival analysis

**Figure 4:**
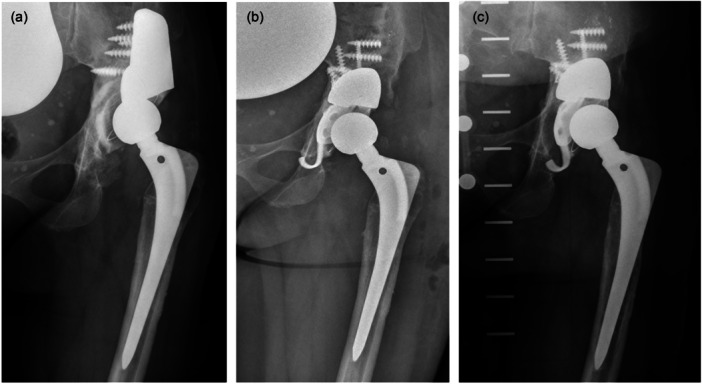
(a) Preoperative anteroposterior radiograph of the hip of a 50‐year‐old patient with severe acetabular bone loss due to aseptic loosening of the cemented cup. (b) Anteroposterior radiograph of the same patient postoperatively, with a modular TM augment in combination with a Ganz reinforcement ring and a cemented PE cup. (c) Anteroposterior radiograph 4.7 years postoperatively, with signs of loosening and dislocation of the reconstruction ring and changed cup position. There are no signs of loosening of the TM augment. TM, trabecular metal.

The cumulative survival rate at 7.4 years with the endpoint ‘revision of the acetabular components for any reason’ was 86.7% (95% confidence interval 56%–96%, number at risk = 10). At the most recent FU two patients (11%) of the study cohort had undergone revision surgery due to aseptic loosening of the acetabular construct (Figure 4) 4.7 years and 5.4 years after surgery, respectively. The original indication for the operation was aseptic loosening of the cup and both patients suffered from a Paprosky type IIIb defect. In both cases, the acetabular implants had to be completely removed and a one‐stage complex acetabular revision was performed.

### Patient‐reported outcome measures

**Figure 5:**
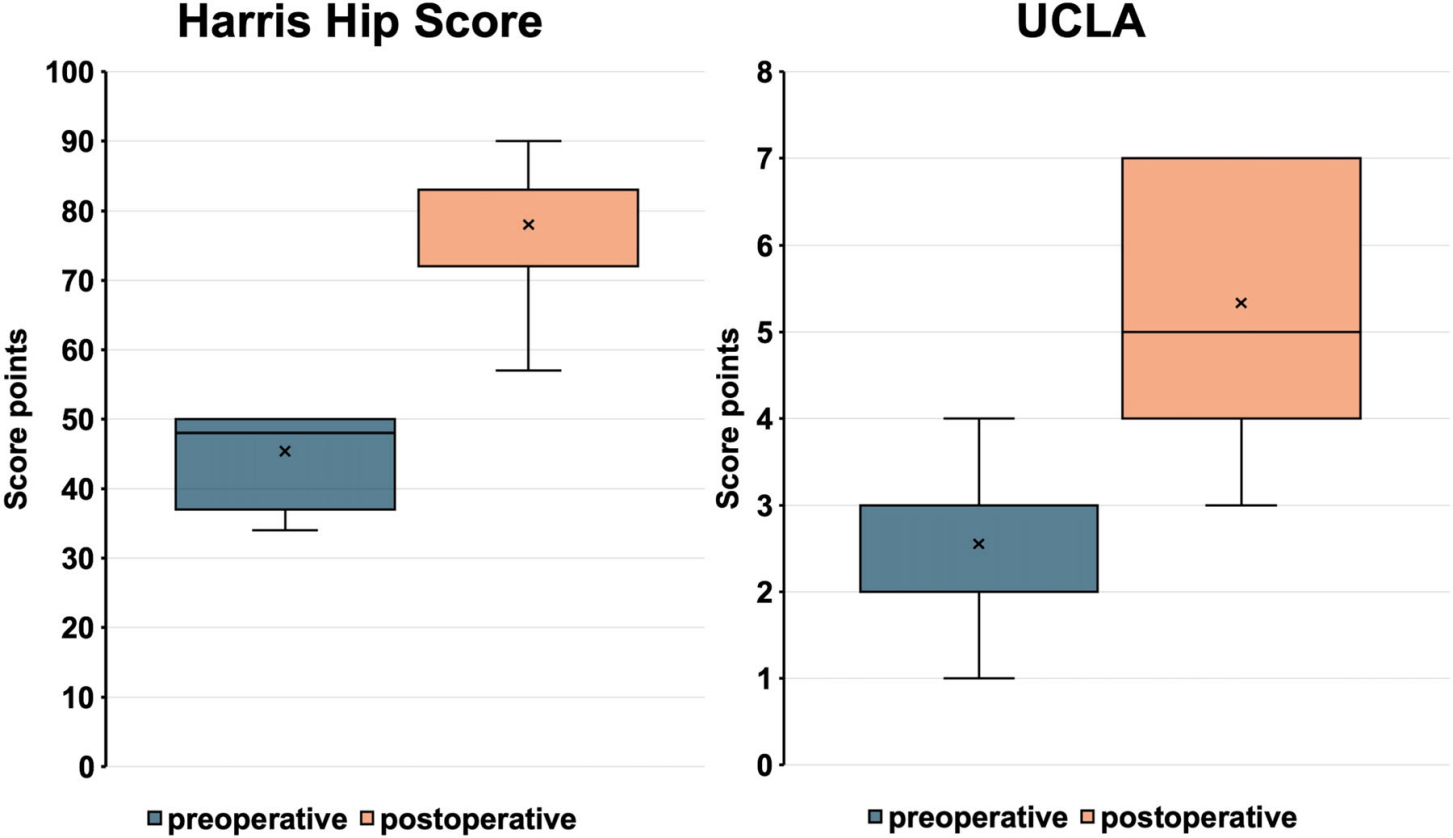
Comparison of Harris Hip Score and UCLA Score before surgery and at last follow‐up, represented in Box–Whisker plots. The box marks the interquartile range, the band inside the box indicates the median, whiskers indicating minimum and maximum data.

Mean HHS improved from 45.4 (SD 17.9; 13.0–85.0) points preoperatively to 78.0 (SD 11.1; 62.0–90.0) points at the last follow‐up (*p* < 0.001). The UCLA Score improved from a mean preoperative score of 2.6 (SD 0.8; 1.0–4.0) points to 5.3 (SD 1.4; 3.0–7.0) points (*p* < 0.001) (Figure 5). Mean postoperative PCS‐12 was 41.4 (SD 9.3; 31.5–55.4) points and MCS‐12 was 49.5 (SD 8.3; 36.9–58.7) points.

### Complications and reoperations

One patient suffered an ischial fracture intraoperatively during the use of a Contourring. The fracture healed without any changes to the aftercare concept. Two patients (11%) of the study cohort had undergone reoperation surgery due to infection (5 days and 8 months after surgery). In both cases, a successful DAIR procedure with replacement of the mobile components was performed. One patient (4.3%) suffered a dislocation 3 days after surgery. In this case, a closed repositioning procedure was performed with a subsequently stable joint and no further dislocation until the last follow‐up.

### Radiographic evaluation

At the last follow‐up, there were no signs of loosening of the modular TM augment due to fully osseointegration of them in all patient. Three patients showed radiolucency lines surrounding the acetabular construct: one patient showed radiolucency lines around the cage without signs of implant migration and without a change of the position of the cup; one patient showed a broken ischial flap with a change of the position of the cup and one patient showed signs of loosening of the reconstruction ring (Ganz reinforcement ring) with a change of the position of the cup into steeper inclination (Figure 4). All of these three patients confirmed absence of pain and clinical symptoms typical for loosening.

### Blood examination

Mean blood tantalum levels were elevated in the study group (0.06 µg/L; SD 0.05, range 0.01–0.13 µg/L), significantly (*p* < 0.001) exceeding those of the control group (0.002 µg/L; SD 0.001, range 0.0002–0.002 µg/L) [30].

## DISCUSSION

Severe acetabular bone defects and pelvic discontinuity are extremely complicated and challenging in revision arthroplasty [34] and adequate primary stability of the acetabular construct is crucial for the long‐term survival of the implant [2]. In the literature, multiple treatment options have been proposed for the management of severe acetabular defects, including the use of porous metal augments with a porous metal acetabular component, standard ilioischial cage reconstruction, a cup‐cage construct with a porous metal acetabular component and an inset cage fixed proximally and distally, bulk acetabular allograft with plating and custom‐made acetabular implants [1, 6, 10]. The best surgical technique has not yet been determined, and none of the solutions mentioned so far have proven to lead to clearly better clinical and radiographic outcomes in the treatment of severe periacetabular bone loss. The combined use of reconstruction rings and modular augments can be a suitable treatment option for selected patients, however, clinical data on the outcome of this surgical technique is scarce. In this study, a combined treatment using a reconstruction ring with a modular TM augment was implemented for individual reconstruction of large acetabular bone defects. This study provides important insights into this procedure, as the mid‐term follow‐up data of our study showed acceptable survival rates and clinical results.

In our cohort, there was a cumulative survival rate of 86.7% for the endpoint ‘revision of the acetabular component for any reason’ at a mean follow‐up of 7.4 years and a significant improvement in PROMs at mid‐term follow‐up. Two patients of the study cohort have had revision surgery due to aseptic loosening of the acetabular reconstruction ring. There was no case of aseptic loosening of the TM augment. In our cohort, radiological follow‐up revealed radiolucencies at the reconstruction ring–bone interface in three of the nine patients assessed. Although these patients remained asymptomatic, such cases should be monitored closely in order to detect increasing loosening or implant migration at an early stage.

These findings align with existing literature on other reconstruction and anti‐protrusion devices, although comparison is hampered by the multitude of reconstruction devices, techniques and definitions of failure used in the literature. Villatte et al. reported a survival rate of 96.2% after 10 years in the reconstruction of severe acetabular defects with morsellized virus‐inactivated bone allograft and reinforcement ring [32], which is slightly higher than in our group. In 3.4% of the cases, a revision was performed due to aseptic loosening and infection. In 19.7% of the cases, radiolucent lines surrounding the cup was seen on X‐ray, without clinical relevance [32]. Corresponding signs were also seen in our cohort, even without clinical symptoms. Makita et al. reported a 15.2‐year survival rate of 85.1% for revision THA using bulk allograft and reinforcement device [22], which is comparable to our results. Using a Mueller reinforcement device, Schlegel et al. [28] found 5‐year survival rates of 95%, as opposed to 64% obtained by Bonnomet et al. [4]. For the Kerboull and Burch‐Schneider devices, the results are equally diverse, with survival rates ranging from 53% to 92.1% for follow‐ups of up to 18.9 years [4, 13, 19, 26]. Overall, loosening of the acetabular components appears to be the most common reason for revision surgery [22, 32], which is confirmed by our study.

An alternative treatment option for patients with massive periacetabular bone loss is the use of custom‐made acetabular components [9]. Custom‐made implants offer an individualized treatment strategy because the hip‐centre, cup orientation and flange geometry can be adapted to the patient's individual geometry for the best possible fixation of the implant on the remaining bone stock [9]. The monoblock structure permits to fill and bridge extensive bone defects hereby reducing a possible source of failure linked to the modularity of off‐the‐shelf revision implants [14] and several clinical studies have shown promising results for this treatment strategy [6, 9]. However, custom‐made implants are not suitable for every patient and the increased costs in comparison to standard implants as well as the delay of the surgery due to long production times [9], which can sometimes take months, are significant disadvantages, especially in the care of elderly and frail patients. In these patients, an immediate surgical treatment and early postoperative mobilization are important in order to avoid further complications, which can be achieved with modular revision systems, such as the use of reconstruction rings in combination with porous metal augments. Furthermore, Froschen et al. report about a high failure rate (23% after 5 years) of custom‐made acetabular implants [9] with periprosthetic joint infection (PJI) as the main complication. A systematic review of the literature confirms the high complication rate, with the main complications being PJI and aseptic loosening as predominant reasons for revision [6]. Possible reasons for this high PJI rate could be the long surgery time, a previous PJI, or the size of the implants [9].

Previous studies have suggested that micromotion at the cement–augment junction may contribute to tantalum particle release. In the context of reinforcement rings, such wear mechanisms could be compounded by complex load transmission and interface dynamics. In our cohort, mean blood tantalum concentrations at mid‐term follow‐up were minor increased (0.06 µg/L) compared to our control group (0.002 µg/L) [30]. To our knowledge, a reference value for serum tantalum concentrations has only been described for a healthy population not exposed to tantalum implants [5, 27] and is specified up to 0.01 µg/L [27]. To our knowledge, no study has investigated blood tantalum concentrations in patients treated with modular tantalum TM augments in combination with a reconstruction ring and a cemented cup. Bruggemann et al. [5] reported elevated blood tantalum concentrations in patients treated with a cementless tantalum cup in primary THA (0.051 µg/L) and in patients who underwent revision surgery with a cementless tantalum revision shell (0.091 µg/L) without the use of tantalum augments after 4 years. Our results demonstrated similar levels of tantalum (0.06 µg/L) in the blood after 7.4 years. Tantalum levels were previously investigated in the blood of patients whose acetabular defects were reconstructed with a TM Augment in combination with either a cemented cup or a cementless TM cup and with slightly higher mean blood tantalum concentrations in these patients of 0.15 and 0.16 µg/L, respectively, compared to the study cohort of this study [30, 31]. Bellouard et al. observed an accumulation of metal particles in various organs, following joint replacement surgery [3]. Tantalum is widely recognized for its high biocompatibility [5, 24]. Experimental data indicate that tantalum oxide nanoparticles can induce apoptosis and stimulate endoplasmic reticulum stress in human endothelial cell cultures [17]. Conversely, Yang et al. [35] reported that tantalum particles caused only minimal cytotoxic and inflammatory effects in THP1 monocytic leukaemia cells, remaining within safe clinical thresholds. Overall, the consequences of significantly increased tantalum concentrations in vivo are not fully understood and require further investigations.

This study is subject to several limitations that should be recognized. It is limited by its retrospective nature and small sample size, which reflects the infrequent use of combined TM augment and reconstruction ring techniques, with only 23 such procedures conducted at our institution over a period of 9 years. Unfortunately, only 9 of the 23 patients could be included in the complete clinical and radiological analysis, which limits the significance of the results. Nonetheless, it provides valuable insights into this specialized and underreported reconstructive strategy by assessing both the radiological and clinical outcomes of a complex individual acetabular defect reconstruction using modular TM augments in combination with a reconstruction ring and cemented revision cups, while also analyzing the concentration of tantalum in the blood of the patients involved.

## CONCLUSION

The reconstruction of extensive acetabular defects during revision THA remains technically demanding. Our findings suggest that combining modular TM augments with acetabular reinforcement rings and cemented cups offers a viable option for individualized reconstruction of severe acetabular defects, with favourable mid‐term outcomes and acceptable complication rates over an average 7.4‐year follow‐up period.

While aseptic loosening was identified as the leading cause of failure, the TM augments demonstrated consistent osseointegration across the cohort. Given the limited feasibility of custom‐made implants in all patients—due to cost, production time, or comorbidity—the modular approach outlined here may represent a valuable and accessible alternative for individualized care in complex revisions.

Further prospective studies with extended follow‐up periods and larger cohorts are needed to validate these findings and to evaluate implant performance beyond the first decade after implantation.

## AUTHOR CONTRIBUTIONS

All authors contributed to the study conception and design. Conceptualization, funding acquisition, material preparation, investigation, project administration and data collection were performed by David Spranz, Lisa‐Marie Müller and Tobias Reiner. Data analysis, visualisation, writing and editing of the manuscript was performed by David Spranz, Lisa‐Marie Müller, Timo Nees, Raphael Trefzer, Pit Hetto, Tilman Walker and Tobias Reiner. The first draft of the manuscript was written by David Spranz, and all authors commented on previous versions of the manuscript. All authors read and approved the final manuscript.

## CONFLICT OF INTEREST STATEMENT

The authors declare no conflicts of interest.

## ETHICS STATEMENT

The Ethics Committee of the Medical University of Heidelberg has approved the study (S‐122/2021). The manuscript does not contain any individual person's data in any form. All patients signed the informed consent form upon entry in the clinical database.

## Data Availability

The datasets used and/or analysed during the current study are available from the corresponding author on reasonable request.
